# Pregnancy as a risk factor for severe influenza infection: an individual participant data meta-analysis

**DOI:** 10.1186/s12879-019-4318-3

**Published:** 2019-08-02

**Authors:** Dominik Mertz, Calvin Ka-Fung Lo, Lyubov Lytvyn, Justin R. Ortiz, Mark Loeb, for the FLURISK-INVESTIGATORS, Li Wei Ang, Mehta Asmita Anlikumar, Isabelle Bonmarin, Victor Hugo Borja-Aburto, Heinz Burgmann, Jordi Carratalà, Gerardo Chowell, Catia Cilloniz, Jessica Cohen, Jeffery Cutter, Laurent Filleul, Shikha Garg, Steffen Geis, Melissa Helferty, Wan-Ting Huang, Seema Jain, Biljana Joves Sevic, Paul Kelly, Gabriela Kusznierz, Nicola Lehners, Luana Lenzi, Ivan T. Ling, Robyn Mitchell, Siobhain A. Mulrennan, Sergio A. Nishioka, Robert Norton, Won Sup Oh, Pablo Orellano, Wolfgang Poeppl, Roberto Pontarolo, Odette Popovici, Alejandro Rodriquez, Bettina Schlehe, Paul Schnitzler, Bunyamin Sertogullarindan, Yuelong Shu, Geoffrey Taylor, Deborah L. Thompson, Antoni Torres, Arianne B. van Gageldonk-Lafeber, Diego Viasus, Quanyi Wang, Cuiling Xu

**Affiliations:** 1 Department of Medicine, McMaster University, Hamilton, ON Canada; 2 Department of Health Research Methods, Evidence and Impact, McMaster University, Hamilton, ON Canada; 3 Departments of Pathology and Molecular Medicine & Clinical Epidemiology and Biostatistics, McMaster University, MDCL 3203, 1200 Main St W, Hamilton, ON L8N 3Z5 Canada; 4 Michael G. DeGroote Institute for Infectious Diseases Research, McMaster University, Hamilton, ON Canada; 5 Initiative for Vaccine Research, World Health Organization, Geneva, Switzerland

**Keywords:** Influenza, Vaccine, Pregnancy, Severity, Meta-analysis

## Abstract

**Background:**

WHO identifies pregnant women to be at increased risk for severe outcomes from influenza virus infections and recommends that they be prioritized for influenza vaccination. The evidence supporting this, however, is inconsistent. Ecologic studies in particular suggest more severe outcomes from influenza infection during pregnancy than studies based on individual patient data. Individual studies however may be underpowered and, as reported in a previous systematic review, confounding factors could not be adjusted for. We therefore conducted an individual participant data meta-analysis to assess the risk for severe outcomes of influenza infection in pregnant women while adjusting for other prognostic factors.

**Methods:**

We contacted authors of studies included in a recently published systematic review. We pooled the individual participant data of women of reproductive age and laboratory confirmation of influenza virus infection. We used a generalized linear mixed model and reported odds ratios (OR) and 95% confidence intervals (CI).

**Results:**

A total of 33 datasets with data on 186,656 individuals were available, including 36,498 eligible women of reproductive age and known pregnancy status. In the multivariable model, pregnancy was associated with a 7 times higher risk of hospital admission (OR 6.80, 95%CI 6.02–7.68), among patients receiving medical care as in- or outpatients, pregnancy was associated with a lower risk of admission to intensive care units (ICU; OR 0.57, 95%CI 0.48–0.69), and was not significantly associated with death (OR 1.00, 95%CI 0.75–1.34).

**Conclusions:**

Our study found a higher risk of influenza associated hospitalization among pregnant women as compared to non-pregnant women. We did not find a higher mortality rate or higher likelihood of ICU admission among pregnant women who sought medical care. However, this study did not address whether a true community based cohort of pregnant women is at higher risk of influenza associated complications.

## Background

Pregnancy is considered to be an important risk factor for severe influenza-associated illness [1–3]. During the 2009 H1N1 influenza pandemic, pregnant women in the United States had high rates of hospitalization; despite representing only 1.0% of the population, pregnant women accounted for 5.8% of the deaths associated with the 2009 H1N1 influenza virus [1, 2]. Citing such disease burden data, favorable influenza vaccine performance, and availability of vaccine delivery platforms globally, the World Health Organization (WHO) has prioritized pregnant women for vaccine receipt [3].

Two systematic reviews conducted by three of the co-authors (DM, JRO, ML), however, questioned whether pregnancy confers an increased risk for severe influenza illness: beyond increasing the need for hospitalization, pregnancy was not associated with more severe influenza associated outcomes in studies where exposure to influenza and outcomes are measured in individuals, including admission to an intensive care unit (ICU), and death [4, 5]. These findings differ from those of ecological studies, where infection by influenza was assumed but not directly measured, most of which suggested more severe outcomes from influenza infection [5]. A critical concern is that prior vaccine exposure, age, underlying health conditions, or antiviral treatments may be different between pregnant women and non-pregnant women of reproductive age hospitalized with influenza, particularly during the 2009 pandemic, which may have confounded the systematic reviews that only analyzed aggregate, study-level data.

To explore the potential influence of confounding, we obtained individual participant-level data (IPD) from studies of reproductive age women with confirmed pregnancy status who had laboratory confirmed influenza virus infection, and conducted a multivariable, IPD meta-analysis to assess the odds of severe influenza outcomes (defined as influenza-associated mortality, ICU and hospital admission) among pregnant women compared to non-pregnant women, adjusting for demographic, comorbid, and clinical covariates.

## Methods

### Search strategy and selection criteria

The eligibility criteria, co-variates of interest and analyses plan were defined a priori.

We requested de-identified IPD from corresponding authors of studies included in our prior systematic reviews, at varying levels of observation, including community, hospital, and ICU [4, 5]. Studies were considered conducted in a ‘community’ setting if participants were seeking health care but have not yet been admitted to a hospital. The search strategy and study selection were reported previously. In short, we searched MEDLINE, CINAHL, Global Health, and the Cochrane Central Register of Controlled Trials from inception up to April 2014 [4, 5]. Ethics approval was obtained where needed by the investigators providing IPD.

Eligible study designs included cohort as well as case control studies published in English, French, Spanish, and German, and must have reported IPD on pregnancy as a risk factor for influenza-associated mortality (primary outcome) and/or influenza associated hospitalization and/or ICU admission (secondary outcomes). Previous systematic reviews included pneumonia as an outcome, however, given its inconsistent definition and rare reporting, we decided a priori to exclude pneumonia as an outcome of interest [4, 5]. Women with influenza virus infection and of reproductive age (defined as 15–45 years) with known pregnancy status were included in this analysis. Influenza virus infection was confirmed through laboratory tests (pre−/post-season or acute−/convalescent serology, viral culture, nucleic acid amplification testing, or influenza antigen detection).

### Data extraction and quality assessment

All datasets were compared with the published results and checked for missing or potentially invalid data. Discrepancies were discussed with the study authors. Study quality was assessed independently and in duplicate using the Newcastle-Ottawa Scale [6] as previously reported [4, 5]. We did not exclude studies based on study quality.

### Data synthesis and analysis

For the primary analyses, we only considered covariates with less than 20% missing data across all studies (‘core variables’). We chose the 20% threshold to balance between excluding potentially relevant risk factors from the multivariable model with excessive missing data, but simultaneously preserving sample size at both the participant and study level. These ‘core variables’ were: age, antiviral usage, diabetes mellitus, cardio-respiratory diseases, immunocompromised status, and influenza vaccination status as defined in the original studies. Vaccination status was only included for ICU admission in a sensitivity analysis due to more than 20% missing data. We conducted a one-stage IPD meta-analysis. First, we run univariate analyses using a generalized linear mixed model (GLMM) with the participant level as a fixed effect and the study level as a random effect. We calculated odds ratios (OR) and associated 95% confidence intervals (CI). For the multivariable analyses, all core variables were included. Furthermore, we conducted post-hoc subgroup analyses separating study populations enrolled in the community and in hospitalized patients, respectively. In a secondary analysis, we added the following covariates with > 20% missing data that had been excluded from the primary analysis but were of potential relevance one by one to the primary model: obesity, smoking status, chronic respiratory diseases alone instead of the composite of cardiac-respiratory co-morbidities, as well as vaccination status for ICU admission. We also considered this as a sensitivity analysis for pregnancy as a risk factor by testing the robustness of our findings when adding additional potential confounders. We did not plan to conduct a subgroup analysis based on influenza season as we anticipated, based on the original systematic review, that there would be sparse data for seasons other than the 2009 H1N1 pandemic. We used PASW Statistics 18 and SAS/STAT 9.4 for analysis. Given the low event rate, we are using the term ‘risk’ throughout the text when discussing ORs to improve readability. Age was treated as a continuous variable and odds were reported per 5-year increase in age.

### Patient and public involvement

There was no patient/public involvement.

## Results

**Fig. 1:**
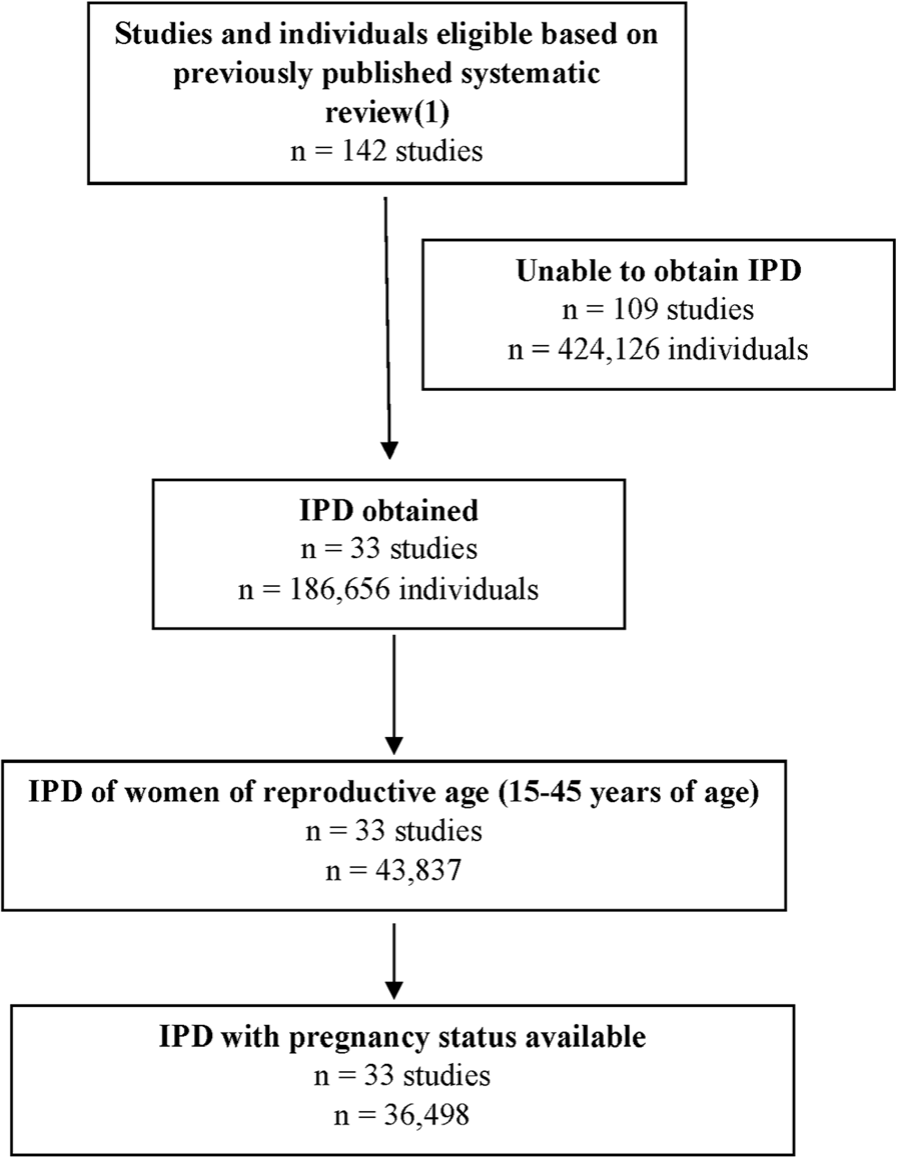
Flow chart Legend: IPD Individual patient data

A total of 33 [7–42] data sets of 142 (23.2%) studies found to be eligible in the previously published systematic reviews were obtained (Fig. 1) [4, 5]. The most common reasons for not being able to obtain IPD were: no response from the authors, or the authors not being able to share the data. We received additional data that were either unpublished, or of which only a subset of the data had been published († in Table 1). Overall, data on 186,656 individuals were available; 31.0% of a total of 31.0% of 610,782 individuals included in the previous systematic review [5]. The average number of eligible participants per study was smaller in our dataset (*n* = 1106) than in studies that had not been shared (*n* = 1685). Otherwise, the study characteristics were similar between included and excluded studies: The median Newcastle-Ottawa score was 6 (interquartile range 6–7) in each group. Similarly, 11/33 (33.3%) of included studies were from low- and middle-income countries as compared to 33/110 (30.0%) in excluded studies, and a cohort study design was the most common design (31 of 33 (93.9%) in included studies compared to 103/110 (93.6%) in excluded studies). Nucleic acid amplification testing was used for the case definition in all studies with data provided for this analysis, with the exception of two studies which used any positive influenza test for case confirmation (e.g. rapid testing, culture) [10, 36]. Studies were conducted in North America, Southern America, Europe, Asia as well as Australia. No studies were available from Africa. Finally, only three datasets included not only patients infected during the 2009 H1N1 pandemic (proportion of US [7–9] and Canadian Public Health data [10] and unpublished data from Nishioka et al. from Brazil).

**Table 1:** Characteristics of 36,498 eligible females 15–45 years of age with known pregnancy status

Author	Eligible females (n)	Pregnant (%)	Mortality (%)	Hospital admission(%)	ICU admission(%)	Country of origin	Study Level^c^
CDC/FluSurv-NET^b^ [7–9]	2531	27.1	2.1	N/A	17.5	USA	Hospital
CNISP^b^ [10]	529	24.4	3.0	N/A	20.6	Canada	Hospital
D’Ortenzio^b^ [11]	17	35.3	47.1	N/A	N/A	Réunion Island	ICU
Echevarria-Zuno [12, 13]	25,206	6.0	1.1	9.9	11.8^a^	Mexico	Community
Fuhrmann^b^ [14]	528	38.4	9.8^a^	N/A	24.2^a^	France	Hospital/ICU
Harris^b^ [15]	51	54.9	2.0^a^	39.2	15.6^a^	Australia	Community
Helferty [16]	889	29.9	7.5^a^	N/A	19.2	Canada	Hospital
Huang [17]	141	7.1	5.7	N/A	24.1	Taiwan	Hospital
Jain [18]	67	26.9	6	N/A	24.2	USA	Hospital
Joves Sevic [19]	14	21.4	21.4	N/A	N/A	Serbia	ICU
Kelly [20]	317	23.3	4.7	N/A	29.3	Australia	Hospital
Kusznierz [21]	48	29.2	39.6	N/A	52.1	Argentina	Hospital
Lehners^b^ [22]	56	51.8	0.0	55.4	10.7	Germany	Community
Lenzi^d^ [23]	488	29.5	15	52.7	N/A	Brazil	Community
Lim^b^ [24]	197	41.1	1.5	N/A	5.6	Singapore	Hospital
Malonda [25]	274	46.4	4.2^a^	86.9	11.1	Spain	Community
Martin-Loeches^b^ [26]	452	20.6	13.9	N/A	N/A	Spain	ICU
Mehta [27]	36	16.7	13.9	N/A	N/A	India	Hospital
Mulrennan [28]	22	22.7	0.0	N/A	22.7	Australia	Hospital
Nishioka^b^	1557	25.8	11.1	N/A	24.6	Brazil	Hospital
Oh [29]	176	11.9	0.6	N/A	4	South Korea	Hospital
Orellano [30]	1689	7.1	2.7	60.5	N/A	Argentina	Community
Poeppl [31]	42	35.7	9.5	69	9.5	Austria	Community
Riquelme^b^ [32]	73	52.1	8.3	72.6	16.4	Spain, Chile	Community
Riquelme^b^ [33]	99	16.2	15.2	N/A	34.7	Global	Hospital
Sertogullarindan [34]	9	22.2	22.2	N/A	N/A	Turkey	ICU
Skarbinski [35]	68	42.6	7.7	N/A	25	USA	Hospital
Thompson [36]	141	35.5	4.3	N/A	21.3	USA	Hospital
Van’t Klooster [38]	339	19.5	0.9	N/A	9.7	Netherlands	Hospital
Viasus [39]	210	46.7	2.4	N/A	10.5	Spain	Hospital
Xu [40]	103	54.4	20.4	N/A	38.6	China	Hospital
Yang^b^ [41]	114	14.9	11.4	93	21.2	China	Community
Zolotusca [42]	15	20.0	13.3	N/A	53.3	Romania	Hospital

^a^Note percentages in Table 1 were based on valid data only; missing data were not included in the calculation

^b^Additional data that was not included in the original publication was provided, unpublished data (Nishioka), and/or several publications based on the dataset provided (CDC, CNISP). FluSurv-NET = Influenza Hospitalization Surveillance Network

^c^Studies were considered conducted in a ‘community’ setting if participants were seeking health care but have not yet been admitted to a hospital

^d^Only a partial dataset available

N/A either no data available or not applicable, e.g. hospital admission as an outcome in a population that was hospitalized as an inclusion criterion for the study. ICU: intensive care unit

We had IPD of 43,837 (23.5%) women of reproductive age (15–45 years of age). Pregnancy status was unavailable for 7339 (16.7%), leaving us with a total of 36,498 eligible participants. Nine studies with a total of 27,993 (76.7%) patients were conducted in a community setting, 20 studies with a total of 8013 (22.0%) enrolled patients that were admitted to the hospital, and 4 studies with 492 (1.3%) patients included patients in an ICU setting, only. Influenza vaccination status was missing in 11.9 and 6.6% of participants with influenza-associated mortality and hospital admission outcomes, respectively, and it was missing in 48.3% of participants with ICU admission as an outcome. Missing data in the other core variables ranged from 7.1 to 19.1% of participants depending on outcome of interest. Among variables for secondary outcomes, obesity status was missing in 36.3 to 54.5%, smoking status in 16.6 to 86.9%, and chronic respiratory comorbidity in 7.4 to 21.1%.

The 4379 pregnant women (12.0%) were significantly younger (mean 26.7 years) compared to non-pregnant women (mean 29.2 years; mean difference 2.43, 95% CI 2.23–2.64) (Table 2). Antiviral treatment (55.4% in pregnant versus 28.7% in non-pregnant women; OR 3.09, 95% CI 2.88–3.31) and receipt of the influenza vaccine (12.1% versus 7.8%; OR 1.62, 95% CI 1.44–1.82) were more common in pregnant women. The prevalence of co-morbidities was similar in both groups with the exception of immunosuppression, which was significantly less common in pregnant women (1.96% versus 2.84%, OR 0.69, 95% CI 0.54–0.87).

**Table 2:** Comparison of pregnant versus non-pregnant participants

	Participant characteristics		
Core model			
	Pregnant (*n* = 4379, 12.0%)	Non-pregnant (*n* = 32,119, 88.0%)	Risk estimate (95% CI)
Mean age (years), SD	26.7 ± 6.1	29.2 ± 8.7	**MD 2.43 (2.23–2.64)**
Antiviral treatment	2073 (55.4%)	8452 (28.7%)	**OR 3.09 (2.88–3.31)**
Vaccinated^a^	367 (12.1%)	2221 (7.8%)	**OR 1.62 (1.44–1.82)**
Diabetes mellitus	150 (3.9%)	1094 (3.8%)	OR 1.03 (0.86–1.22)
Cardio-respiratory	287 (7.7%)	2186 (7.5%)	OR 1.03 (0.91–1.18)
Immunosuppression^c^	74 (2.0%)	814 (2.8%)	**OR 0.69 (0.54–0.87)**
Secondary model			
Obesity	107 (4.3%)	963 (4.7%)	OR 0.92 (0.75–1.13)
Smoking	77 (4.6%)	762 (3.3%)	**OR 1.43 (1.12–1.81)**
Chronic respiratory^b^	251 (6.9%)	1907 (6.6%)	OR 1.05 (0.91–1.20)

Abbreviations: *SD* Standard deviation, *MD* Mean difference, *OR* Odds ratio

^a^Vaccination status not included in the core model for the outcome ICU admission given > 20% missing data

^b^‘Chronic respiratory’ replaced ‘cardio-respiratory’ from the core model in the secondary analysis

^c^‘Immunosuppression’ includes participants with HIV positivity

All variables and figures in bold are indeed statistically significant

### Influenza-associated mortality

All 33 included studies reported on influenza-associated mortality with outcome data available for 36,489 participants. Data from Kusznierz et al. [21] was a subset of a larger dataset by Orellano et al. [30], thus, Kusznierz et al. was excluded. Pregnancy was associated with decreased risk of influenza-associated mortality in univariate analysis (OR 0.66, 95% CI 0.54–0.81; Table 3). Antiviral use and influenza vaccination were also associated with a reduced risk of death (OR 0.77, 95% CI 0.63–0.96 and OR 0.50, 95% CI 0.32–0.78, respectively). Older participants were at a significantly higher risk of influenza-associated mortality (OR 1.24 per 5-year increase in age, 95% CI 1.19–1.29). Participants with diabetes (OR 2.06, 95% CI 1.54–2.75), any cardio-respiratory diseases (OR 1.41, 95% CI 1.11–1.81), and immunocompromised status (OR 2.37, 95% CI 1.77–3.18) were also found to be at higher risk.

**Table 3:** Risk factors for death, hospitalization, and ICU admission in influenza infected women 15–45 years old (core model)

Variable	Univariate analysis			Multivariable analysis^d^	
	Odds ratio (95% CI)	*p*-value	Cases (studies) included	Adjusted odds ratio (95% CI)	*p*-value
Death^a^					
Age (per 5-year increase)	**1.24 (1.19–1.29)**	**< 0.001**	35,591 (32)	**1.19 (1.12–1.26)**	**< 0.001**
Pregnancy	**0.66 (0.54–0.81)**	**< 0.001**	35,591 (32)	1.00 (0.75–1.34)	0.99
Antiviral (yes)	**0.77 (0.63–0.96)**	**0.017**	32,652 (31)	**0.67 (0.49–0.90)**	**0.009**
Vaccination	**0.50 (0.32–0.78)**	**0.002**	31,342 (26)	**0.41 (0.25–0.68)**	**< 0.001**
Cardio-respiratory	**1.41 (1.11–1.81)**	**0.006**	32,167 (31)	1.30 (0.93–1.81)	0.12
Diabetes mellitus	**2.06 (1.54–2.75)**	**< 0.001**	32,442 (31)	**1.67 (1.10–2.51)**	**0.015**
Immunosuppression^e^	**2.37 (1.77–3.18)**	**< 0.001**	31,995 (29)	**2.35 (1.56–3.55)**	**< 0.001**
Hospitalization^b^					
Age (per 5-year increase)	**1.08 (1.06–1.10)**	**< 0.001**	27,972 (9)	**1.12 (1.09–1.15)**	**< 0.001**
Pregnancy	**5.33 (4.79–5.94)**	**< 0.001**	27,972 (9)	**6.80 (6.02–7.68)**	**< 0.001**
Antiviral (yes)	0.96 (0.86–1.07)	0.45	25,814 (9)	1.01 (0.89–1.14)	0.89
Vaccination	0.88 (0.75–1.03)	0.11	27,225 (8)	0.91 (0.76–1.09)	0.31
Cardio-respiratory	**2.24 (1.91–2.62)**	**< 0.001**	26,227 (9)	**2.28 (1.91–2.73)**	**< 0.001**
Diabetes	1.00 (0.78–1.29)	0.99	25,185 (8)	0.92 (0.69–1.23)	0.59
Immunosuppression^e^	1.16 (0.82–1.65)	0.41	25,214 (9)	1.05 (0.69–1.60)	0.83
ICU^c^ Admission^c^					
Age (per 5-year increase)	**1.16 (1.13–1.20)**	**< 0.001**	8836 (26)	**1.07 (1.03–1.11)**	**0.003**
Pregnancy	**0.65 (0.57–0.74)**	**< 0.001**	8836 (26)	**0.57 (0.48–0.69)**	**< 0.001**
Antiviral (yes)	**1.73 (1.48–2.01)**	**< 0.001**	7769 (26)	**1.96 (1.63–2.35)**	**< 0.001**
Cardio-respiratory	**1.39 (1.19–1.63)**	**< 0.001**	7152 (26)	**1.22 (1.01–1.48)**	**0.043**
Diabetes	**1.81 (1.48–2.21)**	**< 0.001**	8019 (26)	**1.58 (1.26–1.99)**	**< 0.001**
Immunosuppression^e^	**1.45 (1.18–1.78)**	**< 0.001**	7516 (24)	1.09 (0.86–1.39)	0.48

^a^Multivariable participants count: 26964, 24 studies, ^b^Multivariable participants count: 23450, 7 studies, ^c^Multivariable participants count: 5766, 24 studies

^d^All variables listed were included in each of the three (death, hospitalization, or ICU admission) multivariable models

^e^‘Immunosuppression’ includes participants with HIV positivity

All variables and figures in bold are indeed statistically significant

In the primary multivariable model, pregnancy was no longer significantly associated with a lower risk of influenza-associated mortality (adjusted OR (aOR) 1.00, 95% CI 0.75–1.34) (Table 3). Antiviral usage (aOR 0.67, 95% CI 0.49–0.90) and vaccination (aOR 0.41, 95% CI 0.25–0.68) remained independently associated. Participants with diabetes (aOR 1.67, 95% CI 1.10–2.51) and those with an immunocompromised status (aOR 2.35, 95% CI 1.56–3.55) remained at a higher risk. Also, each additional 5-year increase in age increased the risk of influenza-associated mortality (aOR 1.19, 95% CI 1.12–1.26).

Similarly, pregnancy was not significantly associated with death in the multivariate post hoc subgroup analyses, whether in community-based (aOR 1.01, 95% CI 0.68–1.51), nor in hospital-based studies (aOR 0.85, 95% CI 0.45–1.59).

Of the additional variables considered in the secondary analysis, obesity was independently associated with an increased risk of influenza-associated mortality (aOR 1.72, 95% CI 1.17–2.52) along with smoking (aOR 1.84, 95% CI 1.04–3.25). The lack of association between pregnancy and influenza-associated mortality persisted when additional covariates were added in the sensitivity analysis, with the exception when smoking status was added to the model resulting in a higher risk for death in pregnant women (aOR 1.62, 95% CI 1.03–2.56) (Table 4).

**Table 4:** Risk factors for death, hospitalization, and intensive-care unit (ICU) admission in influenza infected women 15–45 years old in the secondary and sensitivity analyses

	Death		Hospital admission		ICU admission	
Variable	Adjusted odds ratio (95% CI)	*p*-value	Adjusted odds ratio (95% CI)	*p*-value	Adjusted odds ratio (95% CI)	*p*-value
Obesity	**1.72 (1.17–2. 52)**	**0.005**	**1.49 (1.15–1.93)**	**0.002**	**2.93 (1.99–4.31)**	**< 0.001**
Smoking	**1.84 (1.04–3.25)**	**0.036**	0.93 (0.71–1.22)	0.60	1.56 (0.81–3.00)	0.18
Chronic respiratory	0.92 (0.61–1.37)	0.67	**2.30 (1.92–2.75)**	**< 0.001**	1.20 (0.96–1.49)	0.10
Sensitivity Analysis						
Pregnancy (core model^j^)	1.00 (0.75–1.34)	1.00	**6.80 (6.02–7.68)**	**< 0.001**	**0.57 (0.48–0.69)**	**< 0.001**
Pregnancy (core model including obesity)	0.99 (0.71–1.38)^a^	0.93	**6.83 (6.05–7.71)** ^**d**^	**< 0.001**	0.91 (0.66–1.25)^g^	0.56
Pregnancy (core model including Smoking)	**1.62 (1.03–2.56)** ^**b**^	**0.038**	**7.86 (6.94–8.90)** ^**e**^	**< 0.001**	**0.59 (0.35–0.99)** ^**h**^	**0.047**
Pregnancy (core model including chronic respiratory)	0.99 (0.74–1.32)^c^	0.93	**6.40 (5.58–7.33)** ^**f**^	**< 0.001**	**0.57 (0.47–0.68)** ^**i**^	**< 0.001**

^a^18542, 21 studies, ^b^23064, 7 studies, ^c^26948, 23 studies

^d^17505, 7 studies, ^e^22846, 4 studies, ^f^23438, 6 studies

^g^ 2087, 20 studies, ^h^1069, 11 studies, ^i^5683, 23 studies

^j^Variables included in the core model were age, antiviral use, vaccination (with the exception of ICU admission), cardio-respiratory illness, diabetes, and immunosuppression

All variables and figures in bold are indeed statistically significant

### Influenza-associated hospitalization

Nine of 33 studies (27%) reported on influenza-associated hospital admission with outcome data available for 27,699 participants. Four of the studies were conducted in Europe, two in Australia, and one each in Brazil, China, and Mexico. Pregnant women were at a significantly increased risk for hospitalization compared to non-pregnant women in the univariate analysis (OR 5.33, 95% CI 4.79–5.94) (Table 3). Participants with cardio-respiratory diseases were also more likely to be admitted to the hospital (OR 2.24, 95% CI 1.91–2.62) along with older age (OR 1.08, 95% CI 1.06–1.10 per 5-year increase). No significant associations were found for the other potential risk factors, and antiviral usage and vaccination status were not found to be protective. In the multivariable analysis, pregnancy remained associated with a seven times increase in risk for influenza-associated hospital admission (aOR 6.80, 95% CI 6.02–7.68). The risk increased by 12% (95% CI 1.09–1.15) per 5-year increase in age and any cardio-respiratory diseases were also associated with an increased risk (aOR 2.28, 95% CI 1.91–2.73).

Of the additional variables considered in the secondary analysis, only obesity and chronic respiratory diseases were significantly associated with hospital admission. Pregnancy remained a significant risk factor for hospital admission when these variables were added to the model (Table 4).

### Influenza-associated ICU admission

Data for influenza-associated ICU admission was reported in 26 out of 33 (79%) studies with outcome data available for 9166 participants. The majority of studies were conducted in Europe (*n* = 8, 30.8%), followed by studies from North America (*n* = 7, 26.9%), and Asia (*n* = 5, 19.2%). Pregnancy was associated with a reduced risk for ICU admission in the univariate analysis (OR 0.65, 95% CI 0.57–0.74). Older age by 5-year increase (OR 1.16, 95% CI 1.13–1.20), cardio-respiratory co-morbidities (OR 1.39, 95% CI 1.19–1.63), diabetes (OR 1.81, 95% CI 1.48–2.21), immunosuppression (OR 1.45, 95% CI 1.18–1.78) and antiviral usage (OR 1.73, 95% CI 1.48–2.01) were associated with an increased risk of ICU admission (Table 3).

In the multivariable model, pregnancy remained significantly associated with a decreased risk of ICU admission (OR 0.57, 95% CI 0.48–0.69). The point estimates for the other variables were similar; however, immunosuppression was no longer significantly associated (OR 1.09, 95% CI 0.86–1.39) with ICU admission. In the primary analysis, the comparison group for ICU admission was –depending on data availability- participants who at baseline were admitted to a hospital but not to the ICU (*n* = 309, 4,2% of participants), participants known at baseline to be not admitted to a hospital (*n* = 826, 11.1%) or participants with no information on hospital admission status but information on ICU admission status (*n* = 6298, 84.7%). In our post-hoc subgroup analyses, the association seemed to be driven by studies conducted in hospitalized patients (aOR 0.57, 95% CI 0.46–0.70), while there was no significant association with ICU admission in community-based studies (aOR 0.72, 95% CI 0.42–1.23).

In our secondary analyses, only obesity was associated with significantly increased risk of ICU admission (aOR 2.93, 95% CI 1.99–4.31). When obesity was included in the model, pregnancy was no longer significantly associated (aOR 0.91, 95% CI 0.66–1.25) (Table 4). Vaccination status was not associated with the risk for ICU admission (aOR 0.78, 95% CI 0.59–1.03).

## Discussion

In our IPD meta-analysis, pregnancy was associated with a seven times higher risk of hospitalization but, among patients seeking medical care as in-or outpatients, was not found to be independently associated with influenza-associated mortality, after adjusting for other potential risk factors in multivariable analysis. These findings are consistent with previous systematic reviews/meta-analyses [4, 5]. However, this study could not address whether a true community based cohort of pregnant women is at higher risk of influenza associated complications.

One explanation for our findings is that pregnant women may be more likely to seek care and be preferentially admitted to a hospital because of concerns that they are at higher risk for complications, particularly in the high resource settings where most of the included studies were conducted. The fact that pregnant women were not found to be at increased risk for death or ICU admission despite a higher hospital admission rate would support such an explanation. Similarly, pregnant women being considered to be at higher risk may explain the observation that pregnant women were more likely to be treated with antivirals and were more likely vaccinated. While our multivariable analysis accounted for comorbidities, vaccination status, and antiviral treatment, potential selection bias could not be controlled for. Most of our data were from hospitalized cohorts, a group in which non-severely ill pregnant women may have been overrepresented -if there was a lower threshold to test for influenza and admit women with influenza if pregnant. While precautionary influenza hospitalizations may be preventable with influenza vaccination, this is currently not supported by the available evidence. Furthermore, our data did not provide consistent support for pregnancy being an independent risk factor for severe influenza disease across outcomes. The direction of the association was not consistent among the three outcomes: in the primary analysis, there was a significantly increased risk for hospitalization, a significantly decreased risk for ICU admission, and no significant risk for mortality. These findings are in keeping with previously published systematic reviews [4, 5] and strengthen these findings given the adjustment for individual-level characteristics in this study. It is important to note however, that severe outcomes may not have appeared to be greater in hospitalized pregnant women simply because they were compared to a relatively ill comparison group. However, if pregnant women were admitted to hospital because they were more seriously ill, and not because of a precautionary measure, it is possible that the similar incidence of adverse outcomes would present an increased risk compared to the source population, pregnant women living in the community. Most of our sensitivity analyses were corroborating the findings from the primary analysis, with one notable exception being the multi-variable analysis that included smoking status which suggested a higher mortality rate in pregnant women. This must be interpreted in the light of all other sensitivity analyses corroborating the primary analysis, and the fact that 37% of participants and 79% of studies were excluded from this analysis due to missing data. Of note, there was an association between use of antivirals and a higher likelihood of ICU admission which is most likely due to confounding by indication.

As already outlined in a previous systematic review, the majority of ecological studies suggest more severe outcomes in pregnant women, while a meta-analysis of individual-patient studies did not [5]. The findings in this IPD-meta analysis corroborate the findings of the meta-analysis and contradict most of the ecological studies. As discussed elsewhere, this is most likely related to biases in ecological studies such as use of a population-wide comparator, estimation of pregnancy rates, and lack of tracking of live and still births [5].

Strengths of this review were the extensive quantity of data included along with the breadth of studies and risk factors examined. The IPD allowed us to evaluate pregnancy as an independent risk factor while adjusting for several patient characteristics including comorbidities. The main limitation of our meta-analysis was the potential for selection bias in source studies, where pregnant women enrolled in the studies might have been less ill than non-pregnant women and no studies where women living in the community were followed until hospitalization and afterwards to assess for severe outcomes. Missing data among participants for some covariates was yet another limitation. Furthermore, risk factors may have been defined differently across studies, e.g. the diagnosis of obesity would optimally be based on the body mass index prior to being pregnant. In addition, differences in patient populations resulted in clinical heterogeneity which resulted in statistical heterogeneity as shown in our aggregate data systematic review [5]. Furthermore, it is possible that pregnant women were in general healthier than non-pregnant women, because a minimum level of health is needed to become pregnant. However, the multivariate analysis adjusted for this to the extent possible given the binary data. Data on timing of the antivirals in respect to the outcomes were not available, thus, we are unable to presume causality for any of the associations between antivirals and the clinical outcomes. The risk for severe outcomes may vary by trimester which could not be analyzed given the lack of data available. We were able to obtain 31% of the IPD, only, which could have resulted in a selection bias. However, the study characteristic of in- and excluded studies were similar as were the key findings when compared to the previously published systematic review [5]. An updated search may have identified more studies of potential relevance, but given the time consuming process of obtaining IPD, no update of the literature search while working on this IPD meta-analysis was conducted. Finally, most of the available data were from studies conducted during the 2009 H1N1 pandemic and from high-income countries, thus, the generalizability of our findings to seasonal influenza and low-and middle income countries is unclear.

## Conclusions

Our study found a higher risk of influenza associated hospitalization among pregnant women as compared to non-pregnant women. We did not find a higher mortality rate or higher likelihood of ICU admission among pregnant women who sought medical care as in- or outpatients. However, this study did not address whether a true community based cohort of pregnant women is at higher risk of influenza associated complications. To address this question, a cohort study of pregnant and non-pregnant women with a study population representative of the community who are infected with influenza would need to be conducted.

## Data Availability

The datasets generated and/or analysed during the current study are not publicly available due the various sources of the original data, with some data requiring approval by government agencies. Data can be made available by the corresponding author on reasonable request if approved by co-authors and government bodies where applicable.

## Abbreviations

aOR: Adjusted odds ratio
CI: Confidence interval
GLMM: Generalized linear mixed model
ICU: Intensive care unit
IPD: Individual participant-level data
OR: Odds ratio
US: United States
WHO: World Health Organization
